# Effects of low intraperitoneal pressure and a warmed, humidified carbon dioxide gas in laparoscopic surgery: a randomized clinical trial

**DOI:** 10.1038/s41598-017-10769-1

**Published:** 2017-09-12

**Authors:** Sachiko Matsuzaki, Lise Vernis, Martine Bonnin, Celine Houlle, Aurelie Fournet-Fayard, Giuseppe Rosano, Anne Laure Lafaye, Christian Chartier, Agnes Barriere, Brigitte Storme, Jean-Etienne Bazin, Michel Canis, Revaz Botchorishvili

**Affiliations:** 1 CHU Clermont-Ferrand, Service de Chirurgie Gynécologique, Clermont-Ferrand, France; 2 Université Clermont Auvergne, Institut Pascal, UMR6602, CNRS/UCA/SIGMA, Clermont-Ferrand, France; 3 CHU Clermont-Ferrand, Service d’Anesthésie Réanimation, Clermont-Ferrand, France

## Abstract

Laparoscopic surgery technology continues to advance. However, much less attention has been focused on how alteration of the laparoscopic surgical environment might improve clinical outcomes. We conducted a randomized, 2 × 2 factorial trial to evaluate whether low intraperitoneal pressure (IPP) (8 mmHg) and/or warmed, humidified CO_2_ (WH) gas are better for minimizing the adverse impact of a CO_2_ pneumoperitoneum on the peritoneal environment during laparoscopic surgery and for improving clinical outcomes compared to the standard IPP (12 mmHg) and/or cool and dry CO_2_ (CD) gas. Herein we show that low IPP and WH gas may decrease inflammation in the laparoscopic surgical environment, resulting in better clinical outcomes. Low IPP and/or WH gas significantly lowered expression of inflammation-related genes in peritoneal tissues compared to the standard IPP and/or CD gas. The odds ratios of a visual analogue scale (VAS) pain score >30 in the ward was 0.18 (95% CI: 0.06, 0.52) at 12 hours and 0.06 (95% CI: 0.01, 0.26) at 24 hours in the low IPP group versus the standard IPP group, and 0.16 (95% CI: 0.05, 0.49) at 0 hours and 0.29 (95% CI: 0.10, 0.79) at 12 hours in the WH gas group versus the CD gas group.

## Introduction

Laparoscopic surgery technology has evolved dramatically over the past 3 decades, and continues to advance. However, much less attention has been focused on how alteration of the laparoscopic surgical environment might improve clinical outcomes.

We previously investigated the impact of IPP during a CO_2_ pneumoperitoneum on expression levels of 84 genes known to encode extracellular matrix and adhesion molecules and 84 genes that encode inflammatory cytokine signaling molecules in peritoneal tissues using two real-time polymerase chain reaction (PCR)-based assay panels^1, 2^. We hypothesized that a high IPP during a CO_2_ pneumoperitoneum might adversely affect gene expression of extracellular matrix, adhesion and inflammatory cytokine signaling molecules in peritoneal tissues^3, 4^, if the high IPP caused higher rates of peritoneal dissemination, peritoneal tissue hypoxia and post-operative adhesion formation, as demonstrated in animal studies^5–8^. We identified several differentially expressed genes (4 adhesion-formation-related genes, 4 inflammation-related genes, and 5 hyaluronan [HA]-related genes) in peritoneal tissues in the standard IPP (12 mmHg) group compared with the low IPP (8 mmHg) group. Our previous findings suggested that a low IPP (8 mmHg) might minimize the adverse impacts of IPP on the fibrinolytic system, inflammation, peritoneal fibrosis, and generation of hyaluronan (HA) fragments^1, 2^. However, our previous studies were not randomized^1, 2^. In addition, we did not evaluate whether a low IPP (8 mmHg) could improve clinical outcomes^1, 2^. A Cochrane review concluded that no evidence is currently available to support the use of a low-pressure pneumoperitoneum in low–anesthetic-risk patients undergoing elective laparoscopic cholecystectomy^9^. Regarding temperature and humidity, previous animal and *in vitro* experiments demonstrated that a cool and dry CO_2_ (CD) gas, which we use in a clinical setting, might adversely affect the surgical peritoneal environment^10–13^. However, a Cochrane review concluded that during laparoscopic abdominal surgery, heated gas insufflation, with or without humidification, minimally impacts patient outcomes^14^. However, no studies have yet evaluated the impact of the combined use of a warmed, humidified CO_2_ (WH) gas pneumoperitoneum and low IPP during laparoscopic surgery on the peritoneal environment and postoperative clinical outcomes.

In the present study, we hypothesized that combined use of low IPP and WH gas may be better for minimizing the adverse impact of a CO_2_ pneumoperitoneum on the surgical peritoneal environment during laparoscopic abdominal surgery and improving postoperative clinical outcomes, compared to the standard IPP and CD gas. To test this hypothesis, we conducted the present randomized, 2 × 2 factorial trial.

The primary objective of this study was to compare the impact of low IPP (8 mmHg) versus standard IPP (12 mmHg), and CD gas versus WH gas, on expression levels of 12 genes (4 adhesion-formation-related genes, 4 inflammation-related genes, and 4 hyaluronan [HA]-related genes) in peritoneal biopsy specimens according to our previous studies^1, 2^. Secondary objectives were to compare the impacts of low IPP versus standard IPP, and CD gas versus WH gas, on the quality of postoperative recovery, postoperative pain, intraoperative core body temperature, and intraoperative and postoperative complications.

## Methods

The study was designed as a prospective, 2 × 2 factorial, four–parallel-group, single-center, single-blinded (patients), superiority randomized trial. Patients were recruited at CHU Clermont-Ferrand from September 2013 through June 2016. The study protocol was approved by the Consultative Committee for Protection of Persons in Biomedical Research (CPP) of the Auvergne (France) region and registered by the competent French authority (ANSM, Saint Denis, France). Informed written consent was obtained from each patient prior to surgery. Methods were carried out in accordance with the approved guidelines and regulations. This trial is registered with ClinicalTrials.gov on 24/06/2013, trial number NCT01887028.

During study recruitment, all patients who underwent laparoscopic sub-total hysterectomy with promontofixation for uterine prolapse were assessed for eligibility to participate by the principal investigators (S.M. and R.B.). The full trial protocol can be found in the Supplement (see Supplementary Methods). After obtaining informed written consent, participants are allocated in a 1:1:1:1 ratio by a remote 24-hour-a-day computer-generated randomization system hosted at the Institute for Medical Informatics, Statistics and Documentation, Medical University of Graz, in Graz, Austria (https://www.randomizer.at/) using an algorithm with BMI (<25 vs. ≥25), height (<160 vs. ≥160 cm), and age (<65 vs. ≥65 years) as minimization covariates. This randomized trial employed a 2 × 2 factorial design, with IPP and types of CO_2_ gas as factors, resulting in four experimental arms: 1) 12-mmHg IPP with CD gas, 2) 12-mmHg IPP with WH gas, 3) 8-mmHg IPP with CD gas, and 4) 8-mmHg IPP with WH gas. After randomization, patients can only be excluded if pathological peritoneal tissues such as adhesions are detected just after insertion of the four trocars, a different surgical technique is needed because the sacral promontory is not clearly identified, IPP is changed during the surgery, conversion to laparotomy, or withdrawal of consent. Enrollment in this study is voluntary and patients are allowed to withdraw at any time.

Patients were blinded for the allocated treatment arm until the end of the study. The nurse anesthetists in the postanesthesia care unit (PACU) and the ward nurses, who evaluated postoperative pain using a visual analogue scale (VAS), were also blinded. The database was submitted for analysis to independent statisticians who were blinded and were neither involved in the trial management nor employed by the trial sponsor.

Anesthetic management was performed by 8 staff anesthesiologists (L.V., M.B., A.F., G.R., A.L., C.C., A.B., B.S.) and anesthesiology residents or nurse anesthetists under their supervision. In the operating room, standard ASA anesthetic monitors were placed. All patients received a standardized general anesthetic consisting of premedication with oral administration of 1 mg/kg hydroxyzine hydrochloride suspension 1 hour preoperatively and induction with target-controlled infusion of 0.2 mcg/kg sufentanil and 3 to 5 mg/kg IV propofol. Two mg/kg IV cisatracurium were used to facilitate tracheal intubation. The patient’s lungs were ventilated with a 40:60 mixture of oxygen to nitrous oxide. Desflurane and target-controlled infusion of sufentanil were added for maintenance. To assure suitable operating conditions, neuromuscular blockade was maintained using cisatracurium. After induction of anesthesia, all patients received intraoperative forced-air warming, which was placed on the patient by the anesthesia staff. Intraoperative core temperature was measured at 15-minute intervals using an esophageal probe. For prevention of postoperative nausea and vomiting, 8 mg IV dexamethasone at the beginning of intervention, and 1 mg IV droperidol 1 and 4 mg IV ondansetron at the end of intervention were administrated. 30 minutes before the end of intervention, all patients received 1 g IV paracetamol and 50 mg IV ketoprofene for prevention of postoperative pain.

This study involved 1 staff surgeon (R.B.) who performed all operations with the assistance of a gynecological surgical resident. Insufflation of CO_2_ gas was performed using a Storz electronic endoflator (Karl Storz Endoscopy & GmbH, Tuttlingen, Germany). CO_2_ gas was warmed to 37 °C and humidified to 98% RH using the Fisher & Paykel MR860 Laparoscopic Humidification System (HumiGard, Fisher & Paykel Healthcare, Auckland, New Zealand). For the groups receiving WH gas, 180 mL sterile water was added to the chamber and the humidifier was switched on. For the groups receiving standard CD gas, sterile water was not added to the chamber and the humidifier was not switched on, and CO_2_ gas was delivered at room temperature (21 °C) and 0% relative humidity

When the IPP reached 15 mmHg, four trocars were inserted, immediately after which the IPP was decreased to 12 or 8 mmHg and then maintained at these levels throughout surgery. For all patients, 5 mL ropivacaine hydrochloride solution (2 mg/mL) were infiltrated around the trocar wounds. All incisions were made after ropivacaine infiltration. In addition, 20 mL ropivacaine solution (2 mg/mL) were infused at the beginning of the operation under the right hemidiaphragm. Laparoscopic sub-total hysterectomy with promontofixation used the same surgical technique previously described by our group was performed^15^. Macroscopically normal peritoneum was collected from the anterior parietal wall at the beginning of surgery and every 60 minutes thereafter as previously described^1, 2^.

All patients received our standardized post-operative pain management. On arrival in the PACU, patients were asked to rate their pain at rest using a VAS. After the initial rating, pain ratings were repeated every 20 minutes during the remainder of the PACU stay. When the pain score was >30 of 100, postoperative pain was treated with an IV bolus of 2 to 3 mg morphine, and then 1 to 2 mg IV every 10 minutes to achieve a pain score ≤30 of 100. Intravenous patient-controlled analgesia (IV-PCA) was prepared using morphine (1 mg/mL) and droperidol (0.05 mg/mL). On arrival in the ward, patients were asked to rate their pain at rest using a VAS. Then, the intensity of postoperative pain at rest and/or on moving was assessed using a VAS every 4 hours until 24 hours postoperatively, then 3 times/day until discharge. All patients received 1 g paracetamol and 50 mg of ketoprofene IV every 6 hours until 24 hours postoperatively. Then, pain was managed using oral paracetamol and ketoprofene.

The quality of postoperative functional recovery was assessed using the QoR-40 questionnaire^16^. The QoR-40 was originally designed to assess recovery in five dimensions 24 h after surgery (emotional state, physical comfort, psychological support, physical independence and pain)^16^. It may be most suitable for use in clinical trials or for inpatients^17^. The QoR-40 was administered four times, the day before surgery (between 7:00 and 8:00 pm, baseline score), 24 hours and 48 hours postoperatively, and at the first postoperative visit (30 days after surgery). Intraoperative and postoperative complications were recorded and postoperative complications were classified according to the Clavien-Dindo classification^18^.

Quality of surgical conditions, including the operative technical difficulty, working space, visibility, and pain experienced by the surgeon such as shoulder pain, backache, and hand/finger joint pain during surgery, was rated by the operating surgeon at the end of each procedure using visual analogue scales consisting of 100-mm lines anchored at both ends with 0 and 100.

mRNA levels of 12 genes (connective tissue growth factor [CTGF], matrix metalloproteinase-9 [MMP-9], plasminogen activator inhibitor-1 [PAI-1], tissue plasminogen activator [tPA], thrombospondin-2 [TSP-2], chemokine (C-X-C motif) ligand 2 [CXCL-2], E-selectin, interleukin-10 [IL-10], hyaluronic acid synthase-1 [HAS-1], HAS-2, HAS-3, and hyaluronidase-1 [Hyal-1]) were measured by quantitative real-time RT-PCR with a Light Cycler (Roche, Mannheim, Germany) as previously described^1, 2^.

### Statistical analysis

The STATA program version 13.1 (StataCorp, College Station, TX, USA) was used for statistical analysis. The power calculation of the present trial was based on our previous studies^1, 2^ and our pilot study. The standard deviation was calculated from these gene expression level results for 12 genes and differences that we considered biologically plausible with a significance level of 0.05; 40 patients for each group enabled a power of 91–95% for each gene.

The global QoR-40 scores and the dimensions of the QoR-40 questionnaire were analyzed using the generalized linear mixed model to allow for repeat measurements over time from each patient. The baseline score was considered as a covariate in the analysis, and three main factors were used in the analysis: IPP, type of CO_2_ gas, and time point. The results were summarized as the mean difference in scores between groups, after adjusting for levels at baseline.

VAS pain scores were grouped into three categories; ≤30, 31–70, and >70. A study showed that grouping VAS scores into categories (≤30, 31–70, and >70) provides greater clinical relevance for comparisons than using the full spectrum of measured values or changes in value, when pain is an outcome measure in postoperative patients^19^. During the PACU stay, the maximum pain score before receiving morphine was assessed at a single time point. Logistic regression was used for the analysis. During the inpatient ward stay, pain scores were measured at multiple time points. To allow for multiple measurements over time, the analysis was performed using mixed logistic regression methods. Two-level models were used, with individual measurements nested within patients. To allow for varying pain scores over time, terms for time were included in the regression model. Linear, squared, and cubic terms for time were all included to allow modelling of a flexible relationship between time and pain score. Interactions between the two treatments (IPP and type of CO_2_ gas) and time were included to allow the treatment effects to vary over the course of the inpatient stay. The regression models were simplified to omit non-significant interactions from the final model. Mixed logistic regression was used for the analysis.

The gene expression levels in peritoneal biopsy specimens relative to levels of a reference gene (GAPDH) were assessed at 0 hours, and at 1 and 2 hours during a CO_2_ pneumoperitoneum. This analysis approach considered the 1- and 2-hour values as separate outcomes, which were thus analyzed separately. Linear regression was used for the analysis, with the baseline (0 hours) values included as a covariate.

For the remaining analyses, groups were compared using Fisher’s exact test for categorical variables, the Mann-Whitney U test for nonparametric continuous variables, and the t test for parametric continuous variables. Statistical significance was accepted at the 0.05 level.

### Data availability

The datasets generated during and/or analysed during the current study are available from the corresponding author on reasonable request.

## Results

**Figure 1:**
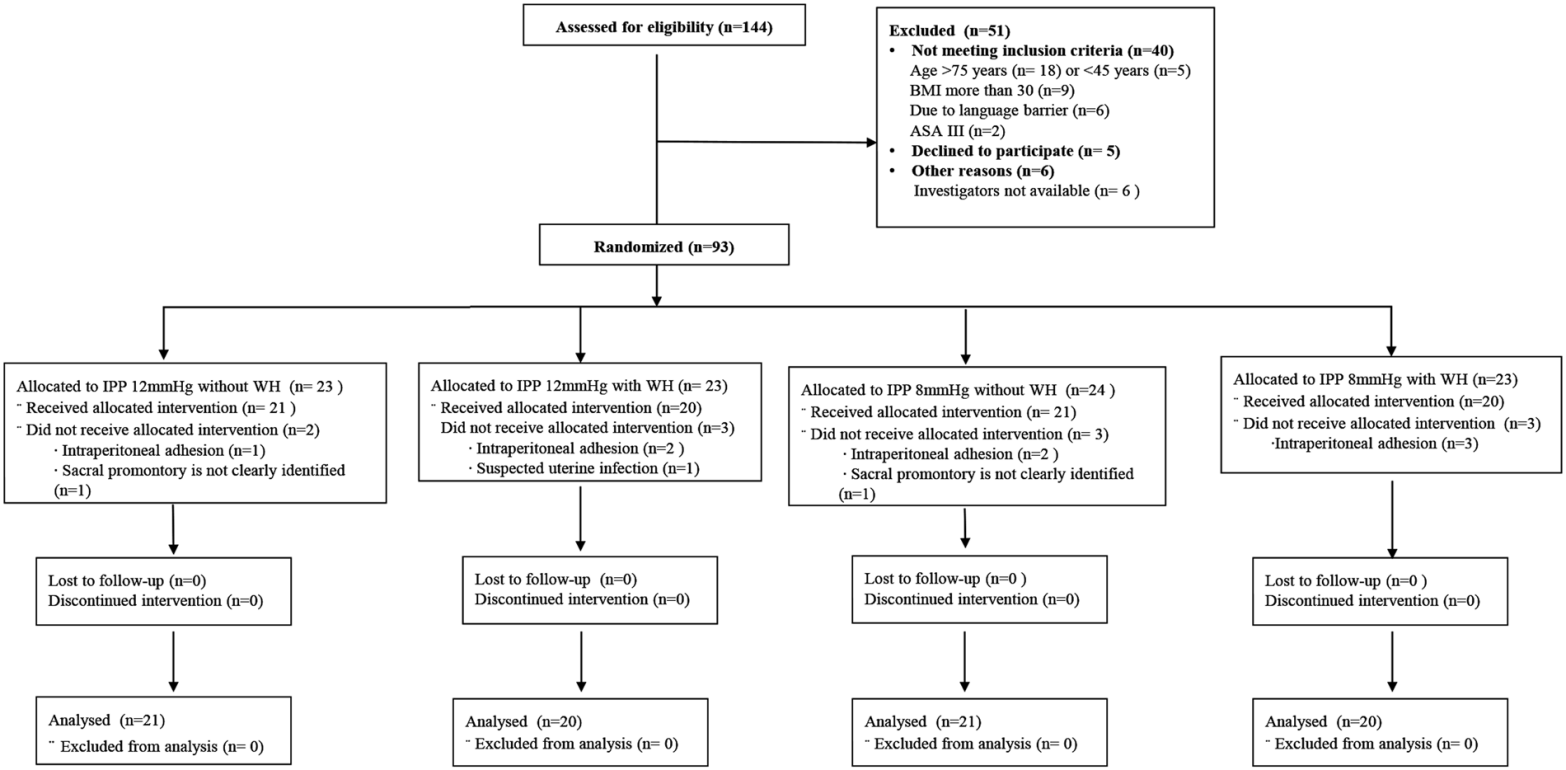
CONSORT diagram.

A total of 144 patients were assessed for eligibility and 93 patients consented and were randomly assigned into the study. Nine patients were excluded after randomization. We therefore collected and analyzed data from 82 patients. The detailed patient flow is shown in the CONSORT diagram in Fig. 1. Patient and surgical characteristics by randomization group and by treatment group are presented in Tables 1 and 2, respectively. Patient and surgical characteristics did not differ between the 12-mmHg and 8-mmHg groups, or between the CD gas and WH gas groups, except for the CO_2_ volume used during CO_2_ pneumoperitoneum between the 12-mmHg and 8-mmHg groups: the CO_2_ volume used in the 8-mmHg group was significantly smaller than that used in the 12-mmHg group (Table 2).

**Table 1:** Baseline Characteristics by Randomization Group.

Variable	12 mmHg	12 mmHg	8 mmHg	8 mmHg
	CD	WH	CD	WH
	(n = 21)	(n = 20)	(n = 21)	(n = 20)
Age, mean (SD), y	64.0 (9.0)	63.6 (7.5)	61.2 (7.7)	60.8 (10.5)
Hight, mean (SD), cm	161.3 (6.2)	160.2 (7.0)	163.0 (4.6)	162.3 (5.4)
Body weight, Mean (SD), kg	61.8 (9.1)	62.0 (8.1)	65.6 (11.1)	64.6 (10.4)
Body mass index, mean (SD)	25.1 (2.9)	24.3(3.0)	24.7 (3.3)	25.1 (2.9)
American Society of Anesthesiologists (ASA) status, No. (%)				
ASA 1	9 (42.9)	5 (25.0)	7 (33.3)	7 (35.0)
ASA 2	12 (57.1)	15 (75.0)	14 (66.7)	13 (65.0)
Basal QoR-40, mean (SD)	191.6 (7.7)	187.5 (9.6)	187.7 (8.3)	186.0 (10.4)
Additional surgical procedure, No. (%)				
None	8 (38.1)	7 (35.0)	8 (38.1)	7 (35.0)
Burch colposuspension (Burch)	12 (57.0)	13 (65.0)	12 (57.1)	12 (60.0)
Tension-free vaginal tape procedure (TVT)	1 (4.8)	0 (0.0)	1 (4.8)	1 (5.0)
Operating time, mean (SD), min				
Total operating time	169.7 (32.3)	173.6 (30.0)	169.0 (28.9)	167.5 (30.1)
Pneumoperitoneum time	154.1 (29.0)	159.8 (24.9)	158.1 (33.9)	153.8 (27.5)
Volume CO_2_ used, mean (SD), L	404.3 (174.1)	365.8 (157.2)	275.8 (123.2)	239.2 (52.9)
Trendelenburg position, mean (SD), degree	22.6 (3.6)	22.3 (4.9)	21.7 (3.9)	25.0 (2.9)
Intraoperative sufentanyl usage, mean (SD), μg	48.4 (7.4)	48.0 (7.8)	49.1 (6.8)	46.3 (6.1)
Core temperature, mean (SD), °C				
Start temperature	36.1 (0.40)	36.0 (0.40)	36.1 (0.36)	36.0 (0.30)
Minimum temperature	35.8 (0.50)	35.8 (0.60)	36.0 (0.50)	35.9 (0.30)
Maximum temperature	36.1 (0.40)	36.0 (0.40)	36.3(0.40)	36.0 (0.30)
Final temperature	36.0 (0.30)	36.0 (0.40)	36.2 (0.50)	36.0 (0.30)
PACU length of stay (LOS), mean (SD), min	115.5 (45.2)	102.5 (31.4)	93.8 (29.5)	104.8 (38.1)
Morphine requirement in the PACU				
Patients requiring morphine, No. (%)	11 (52.4)	13 (65.0)	10 (47.6)	8 (40.0)
Morphine usage, median (IQR), mg	1 (5)	4 (6.5)	3 (6)	0 (4)
Patients requiring morphine in the ward, No. (%)	2 (9.5)	2 (10)	1 (4.8)	0 (0)
Hospital LOS No. (%)				
<48 h	5 (23.8)	7 (35.0)	7 (33.3)	5 (25.0)
<48 h <72 h	14 (66.7)	12 (60.0)	13 (61.9)	15 (75.0)
<72 h <96 h	2 (9.5)	1 (5.0)	1 (4.8)	0 (0.0)

**Table 2:** Baseline Characteristics by Intervention.

Variable	IPP			Type of CO_2_ gas		
	12 mmHg (n = 41)	8 mmHg (n = 41)	P-value	CD (n = 42)	WH (n = 40)	P-value
Age, mean (SD), y	63.8 (8.2)	61.0 (9.1)	0.15	62.6 (8.4)	62.2 (9.1)	0.80
Hight, mean (SD), cm	161.3 (6.2)	161.6 (5.0)	0.77	161.6 (6.0)	161.2 (5.2)	0.78
Body weight, mean (SD), kg	62.6 (9.3)	64.4 (10.2)	0.40	63.7 (10.2)	63.3 (9.3)	0.85
Body mass index, mean (SD)	24.6 (3.4)	24.9(3.1)	0.67	24.9 (3.4)	24.7 (3)	0.82
ASA status, No. (%)						
ASA 1	14 (34.1)	14 (34.1)	1	16 (38.1)	12 (30.0)	0.49
ASA 2	27 (65.9)	27 (65.9)		26 (61.9)	28 (70.0)	
Basal QoR 40, mean (SD)	189.6 (8.8)	186.8 (9.3)	0.18	189.7 (8.1)	186.7 (9.9)	0.15
Additional surgical procedure, No. (%)						
None	15 (36.6)	15 (36.6)	0.93	16 (38.1)	14 (35.0)	0.93
Burch	25 (61.0)	24 (58.5)		24 (57.1)	25 (62.5)	
TVT	1 (2.4)	2 (4.9)		2 (4.8)	1 (2.5)	
Operating time, mean (SD), min						
Total operating time	171.6 (30.7)	168.9 (27.3)	0.60	169.0 (28.9)	170.6 (29.8)	0.81
Pneumoperitoneum time	156.9 (26.8)	157.4 (31.3)	0.92	157.7 (31.9)	156.7 (26.1)	0.88
Volume CO_2_ used, mean (SD), L	385.5 (165.2)	257.9 (96.3)	**0**.**0001**	340.0 (162.6)	302.5 (132.3)	0.26
Trendelenburg position, mean (SD), degree	22.4 (4.2)	23.3 (3.8)	0.30	22.1 (3.7)	23.7 (4.2)	0.08
Intraoperative sufentanyl usage, mean (SD), μg	48.5 (7.2)	47.7 (6.5)	0.60	48.8 (7.0)	47.1 (7.0)	0.29
Core temperature, mean (SD), °C						
Start temperature	36.0 (0.38)	36.0 (0.35)	0.60	36.1 (0.36)	36.0 (0.34)	0.73
Minimum temperature	35.8 (0.52)	35.9 (0.39)	0.22	35.8 (0.45)	35.9 (0.48)	0.68
Maximum temperature	36.0 (0.40)	36.1 (0.41)	0.20	36.0 (0.37)	36.2 (0.43)	0.32
Final temperature	36.0 (0.33)	36.1 (0.41)	0.38	36.1 (0.42)	36.0 (0.32)	0.54
PACU LOS, mean (SD), min	109.4 (38.5)	98.7 (34.4)	0.19	104.4 (38.6)	103.7 (35.1)	0.92
Morphine requirement in the PACU						
Patients requiring morphine, No. (%)	24 (58.5)	18 (43.9)	0.20	21 (50.0)	21 (52.5)	0.83
Morphine usage, median (IQR), mg	2 (6)	0 (5)	0.41	3 (5)	0.5 (5)	0.83
Patients requiring morphine in the ward, No. (%)	4 (9.8)	1 (2.4)	0.36	3 (7.1)	2 (5.0)	1
Hospital LOS, No. (%)						
<48 h	12 (29.3)	12 (29.3)	0.79	12 (28.6)	12 (30.0)	0.74
<48 h <72 h	26 (63.4)	28 (68.3)		27 (64.3)	27 (67.5)	
<72 h <96 h	3 (7.3)	1 (2.4)		3 (7.1)	1 (2.5)	

### Gene expression in peritoneal biopsy specimens

Results are shown in Table 3.

**Table 3:** Gene expression in peritoneal biopsy specimens.

Gene	Timepoint	Variable	Subgroup^a^	Ratio or difference^b^ (95% CI)	P-value
**Adhesion-formation related genes**					
	**CTGF**				
	1 hour	IPP^c^	—	0.66 (0.48, 0.92)	**0**.**02**
		CO_2_ gas^d^	—	1.00 (0.72, 1.39)	0.99
	2 hours	IPP^c^	CD	0.49 (0.31, 0.78)	**0**.**003**
			WH	1.16 (0.72, 1.87)	0.54
		CO_2_ gas^d^	12 mmHg	0.53 (0.34, 0.84)	**0**.**008**
			8 mmHg	1.25 (0.78, 2.03)	0.35
	**MMP-9**				
	1 hour	IPP^c^	—	0.29 (0.21, 0.40)	**<0**.**001**
		CO_2_ gas^d^	—	0.81 (0.58, 1.12)	0.20
	2 hours	IPP^c^	CD	0.18 (0.10, 0.30)	**<0**.**001**
			WH	0.93 (0.53, 1.62)	0.79
		CO_2_ gas^d^	12 mmHg	0.19 (0.11, 0.33)	**<0**.**001**
			8 mmHg	1.02 (0.58, 1.80)	0.95
	**PAI-1**				
	1 hour	IPP^c^	—	0.41 (0.29, 0.58)	**<0**.**001**
		CO_2_ gas^d^	—	1.14 (0.81, 1.59)	0.45
	2 hours	IPP^c^	—	0.33 (0.23, 0.48)	**<0**.**001**
		CO_2_ gas^d^	—	0.62 (0.43, 0.89)	**0**.**01**
	**tPA**				
	1 hour	IPP^c^	—	0.92 (0.68, 1.26)	0.60
		CO_2_ gas^d^	—	1.04 (0.76, 1.42)	0.80
	2 hours	IPP^c^	—	0.91 (0.63, 1.32)	0.62
		CO_2_ gas^d^	—	0.77 (0.53, 1.12)	0.17
**Inflammation related-genes**					
**Pro-inflammatorygenes**					
	**CXCL-2**				
	1 hour	IPP^c^	—	0.94 (0.69, 1.27)	0.67
		CO_2_ gas^d^	—	0.86 (0.63, 1.17)	0.34
	2 hours	IPP^c^	CD	0.27 (0.17, 0.42)	**<0**.**001**
			WH	0.60 (0.39, 0.93)	**0**.**02**
		CO_2_ gas^d^	12 mmHg	0.56 (0.37, 0.87)	**0**.**01**
			8 mmHg	1.25 (0.80, 1.96)	0.32
	**E-selectin**				
	1 hour	IPP^c^	—	0.53 (0.39, 0.73)	**<0**.**001**
		CO_2_ gas^d^	—	0.83 (0.61, 1.13)	0.23
	2 hours	IPP^c^	CD	0.19 (0.11, 0.33)	**<0**.**001**
			WH	0.43 (0.25, 0.73)	**0**.**002**
		CO_2_ gas^d^	12 mmHg	0.27 (0.16, 0.46)	**<0**.**001**
			8 mmHg	0.60 (0.35, 1.05)	0.07
**Anti-inflammatory genes**					
	**IL-10**				
	1 hour	IPP^c^	—	0.67 (0.44, 1.01)	0.05
		CO_2_ gas^d^	—	0.94 (0.62, 1.45)	0.78
	2 hours	IPP^c^	CD	4.22 (2.54, 7.02)	**<0**.**001**
			WH	0.93 (0.56, 1.56)	0.79
		CO_2_ gas^d^	12 mmHg	4.46 (2.69, 7.38)	**<0**.**001**
			8 mmHg	0.99 (0.59, 1.66)	0.96
	**TSP2**				
	1 hour	IPP^c^	—	−8 (−33, 16)	0.52
		CO_2_ gas^d^	—	12 (−12, 35)	0.34
	2 hours	IPP^c^	CD	66 (26, 106)	**0**.**001**
			WH	8 (−32, 48)	0.69
		CO_2_ gas^d^	12 mmHg	66 (27, 104)	**0**.**001**
			8 mmHg	8 (−32, 47)	0.71
**HA related genes**					
	**HAS-1**				
	1 hour	IPP^c^	—	1.67 (1.24, 2.24)	**0**.**001**
		CO_2_ gas^d^	—	1.57 (1.17, 2.12)	**0**.**003**
	2 hours	IPP^c^	CD	2.92 (1.59, 5.38)	**0**.**001**
			WH	1.23 (0.67, 2.25)	0.50
		CO_2_ gas^d^	12 mmHg	3.90 (2.15, 7.08)	**<0**.**001**
			8 mmHg	1.63 (0.88, 3.03)	0.12
	**HAS-2**				
	1 hour	IPP^c^	—	1.07 (0.79, 1.45)	0.67
		CO_2_ gas^d^	—	1.44 (1.06, 1.96)	**0**.**02**
	2 hours	IPP^c^	—	1.64 (1.12, 2.40)	**0**.**01**
		CO_2_ gas^d^	—	2.15 (1.47, 3.14)	**<0**.**001**
	**HAS-3**				
	1 hour	IPP^c^	CD	2.82 (1.84, 4.32)	**<0**.**001**
			WH	1.28 (0.83, 1.97)	0.27
		CO_2_ gas^d^	12 mmHg	2.83 (1.84, 4.35)	**<0**.**001**
			8 mmHg	1.28 (0.83, 1.97)	0.26
	2 hours	IPP^c^	CD	3.32 (2.10, 5.25)	**<0**.**001**
			WH	1.56 (0.99, 2.47)	0.06
		CO_2_ gas^d^	12 mmHg	3.61 (2.30, 5.67)	**<0**.**001**
			8 mmHg	1.70 (1.07, 2.70)	**0**.**03**
	**Hyal-1**				
	1 hour	IPP^c^	—	0.67 (0.52, 0.87)	**0**.**003**
		CO_2_ gas^d^	—	0.62 (0.48, 0.80)	**<0**.**001**
	2 hours	IPP^c^	CD	1.15 (0.82, 0.61)	0.41
			WH	0.70 (0.50, 0.98)	**0**.**04**
		CO_2_ gas^d^	12 mmHg	1.12 (0.81, 1.55)	0.48
			8 mmHg	0.72 (0.52, 0.99)	**0**.**04**

^a^When significant interactions were observed between the type of CO_2_ gas and IPP, the effects of IPP and type of CO_2_ gas were summarised separately for each subgroup of the other variable.

^b^Due to the skewed distribution of the outcome values of CTGF, MMP9, PAI-1, tPA, CXCL-2, E-selectin, IL-10, HAS-1, HAS-2, HAS-3, and Hyal-1 expression, expression levels of these genes were analyzed on a log scale. The results are summarised as the ratio of expression level between groups, after adjusting for levels at baseline (0 hours). An examination of TSP-2 gene expression levels suggested they had an approximately normally distribution. Due to the analysis on the original scale, the regression coefficients from the regression analyses were reported. These represent the mean difference in expression levels between groups, after adjusting for levels at baseline (0 hours).

^c^When comparing the 8-mmHg group relative to the 12-mmHg group.

^d^When comparing the WH group relative to the CD group.

Number of peritoneal tissues.

12-mmHg group: n = 41 at 0 hours (baseline), n = 41 at 1 hour, n = 36 at 2 hours.

8-mmHg group: n = 41 at 0 hours (baseline), n = 41 at 1 hour, n = 34 at 2 hours.

CD group: n = 42 at 0 hours (baseline), n = 42 at 1 hour, n = 35 at 2 hours.

WH group: n = 40 at 0 hours (baseline), n = 40 at 1 hour, n = 35 at 2 hours.

#### Adhesion-formation–related genes

At 1 hour, a significant effect of IPP on expression of CTGF, MMP 9, and PAI-1 was observed. At 2 hours, significant effects of IPP and type of CO_2_ gas on CTGF, MMP 9, and PAI-1 expression were observed.

#### Inflammation-related genes

At 1 hour, no significant effect of either IPP or type of CO_2_ gas was observed on CXCL-2, E-selectin, IL-10 expression or TSP2. At 2 hours, significant effects of IPP and type of CO_2_ gas on these four genes were observed.

#### Hyaluronic acid (HA)-related genes

At 1 hour, a significant effect of IPP and/or type of gas was observed on HAS-1, HAS-2, HAS-3, and Hyal-1 expression. At 2 hours, significant effects of IPP and type of CO_2_ gas were observed on HAS-1, HAS-3, HAS-2, and Hyal-1 expression.

### Quality of postoperative recovery

Results are shown in Table 4. No differences in basal global QoR-40 scores were observed between the 12-mmHg and 8-mmHg groups, or between the WH gas and CD gas groups (Table 2). For all outcomes (global, five dimensions), no statistically significant interactions were observed between IPP and type of CO_2_ gas. For the global QoR-40 score and for the four dimensions of the QoR-40, “emotional state,” “physical comfort,” “physical independence,” and “psychological support,” no significant time by IPP interaction or time by type of CO_2_ gas interaction was observed. Global QoR-40 scores were higher in the 8-mmHg group than in the 12-mmHg group (mean difference: 1.4, p = 0.006). No significant differences in global QoR-40 scores were observed between the CD gas and WH gas groups. “Psychological support” was significantly higher in the 8-mmHg group than in the 12-mmHg groups (p = 0.04). However, the mean difference was only 0.07 between groups. For the “pain” dimension, a significant time by IPP interaction was observed (p = 0.008), but no significant time by type of CO_2_ gas interaction was noted. The score for the “pain” dimension was significantly higher in the 8-mmHg group than in the 12-mmHg group at 24 hours (mean difference: 2.3, p < 0.001) and at 48 hours (mean difference: 1.6, p = 0.008) postoperatively. No significant difference in “pain” was observed between the 8-mmHg and 12-mmHg groups 30 days postoperatively. Furthermore, no significant difference in “pain” was observed between the CD gas and WH gas groups.

**Table 4:** QoR-40 Scores.

Outcome	Variable	Subgroup^a^	Difference^b^ Mean (95% CI)	P-value
Global score	IPP^c^	—	1.4 (0.4, 2.5)	**0.006**
	CO_2_ gas^d^	—	−0.1 (−1.1, 0.9)	0.85
C1: Emotional	IPP^c^	—	0.5 (−0.1, 1.1)	0.12
State	CO_2_ gas^d^	—	0.4 (−0.2, 1.0)	0.17
C2: Physical	IPP^c^	—	0.1 (−0.1, 0.4)	0.38
Comfort	CO_2_ gas^d^	—	0.0 (−0.2, 0.3)	0.72
C3: Psychological	IPP^c^	—	0.07 (0.05, 0.13)	**0.04**
Support	CO_2_ gas^d^	—	0.02 (−0.05, 0.08)	0.60
C4: Physical	IPP^c^	—	0.12 (0.00, 0.23)	0.06
Independence	CO_2_ gas^d^	—	−0.08 (−0.20, 0.04)	0.17
C5: Pain	IPP^c^	24 hours	2.3 (1.1, 3.5)	**<0.001**
		48 hours	1.6 (0.4, 2.8)	**0.008**
		30 days	0.3 (−0.1, 0.7)	0.12
	CO_2_ gas^d^	—	−0.2 (−0.6, 0.2)	0.23

^a^For the “pain” dimension, a significant time by IPP interaction was observed. Thus, the effect of IPP was quantified at each timepoint. ^b^Differences in the QoR-40 were analyzed using the generalized linear mixed model. ^c^When comparing the 8-mmHg group relative to the 12-mmHg group. ^d^When comparing the WH group relative to the CD group.

Number of patients.

12-mmHg group: n = 41 (baseline), n = 41 at 24 hours, n = 29 at 48 hours, n = 41 at 30 days.

8-mmHg group: n = 41 (baseline), n = 41 at 24 hours, n = 28 at 48 hours, n = 41 at 30 days.

CD group: n = 42 (baseline), n = 42 at 24 hours, n = 31 at 48 hours, n = 42 at 30 days.

WH group: n = 40 (baseline), n = 40 at 24 hours, n = 26 at 48 hours, n = 40 at 30 days.

### VAS pain score

There were no patients with a VAS score >30 the day before surgery (between 7:00 and 8:00 pm, baseline score). In the present analysis, we only analyzed VAS pain scores at rest, because during the early postoperative period up to 12 hours, many patients stayed at rest; thus, pain scores on movement could not be sufficiently evaluated during this period. No patients had shoulder pain. We have been systematically performing infusion of ropivacaine solution under the right hemidiaphragm for over 10 years. During this time, we have had no or few complaints about shoulder pain after laparoscopic surgery.

In the present study, there were very few patients with a VAS score >70 postoperatively. Thus, pain scores were categorized as either ≤30 (no or mild pain) or >30 (clinically relevant moderate to severe pain), giving a binary measure^19^.

In the PACU, no significant differences in the number of patients who required morphine and morphine dose infused were observed between the 12-mmHg IPP and 8-mmHg IPP groups or between the WH gas and CD gas groups (Table 2) (Table 5). No significant interaction in VAS pain scores was observed between IPP and type of CO_2_ gas (p = 0.81). Neither IPP nor the type of gas was associated with the likelihood of a high VAS pain score >30 in the PACU (p = 0.81 and p = 0.70, respectively) (Table 5).

**Table 5:** Odds ratios of a VAS pain score of >30 in the PACU.

Variable	Category	No. of patients	Score >30 , N (%)	Odds Ratio (95% CI)	P-value
IPP	12 mmHg	41	29 (71)	1	0.81
	8 mmHg	41	28 (68)	0.89 (0.35, 2.29)	
Type of CO_2_ gas	CD	42	30 (71)	1	0.70
	WH	40	27 (68)	0.83 (0.32, 2.13)	

**Figure 2:**
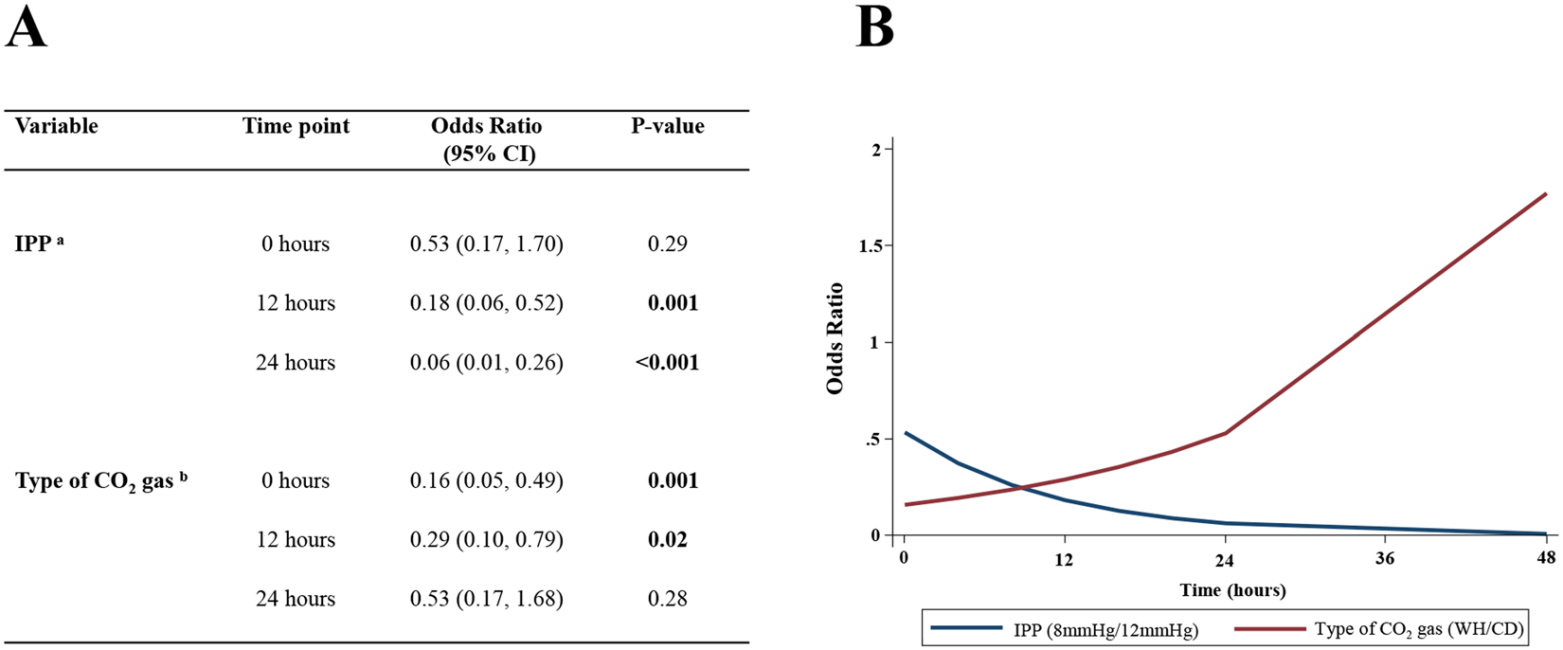
Odds ratios (**A** and **B**) of a VAS pain score of >30 in the ward. ^a^Odds ratios when comparing the 8-mmHg group relative to the 12-mmHg group. ^b^Odds ratios when comparing the WH group relative to the CD group. Number of patients. 12-mmHg group: n = 41 at 0 hours, n = 41 at 12 hours; n = 41 at 24 hours. 8-mmHg group: n = 41 at 0 hours, n = 41 at 12 hours; n = 41 at 24 hours. CD group: n = 42 at 0 hours, n = 42 at 12 hours; n = 42 at 24 hours. WH group: n = 40 at 0 hours, n = 40 at 12 hours; n = 40 at 24 hours.

In the ward, few patients required morphine injections by a PCA (Tables 1 and 2). A non-significant three-way interaction was observed between IPP, type of CO_2_ gas and time (p = 0.21), and a non- significant-two-way interaction was observed between IPP and type of CO_2_ gas (p = 0.22). No significant difference was observed in the likelihood of a VAS pain score >30 between the 8-mmHg and 12-mmHg groups at 0 hours (p = 0.29) (Fig. 2A and B, Table 6). However, there was a significant difference between the 8-mmHg and the 12-mmHg groups was observed at 12 hours (p = 0.001) and at 24 hours (p < 0.001) (Fig. 2A and B, Table 6). The odds ratios of a VAS pain score > 30 was 0.18 (95% CI: 0.06, 0.52) at 12 hours and 0.06 (95% CI: 0.01, 0.26) at 24 hours when comparing the 8-mmHg group relative to the 12-mmHg group (Fig. 2A and B, Table 6). In addition, significant differences in in the likelihood of a VAS pain score > 30 were observed at 0 hours (p = 0.001) and at 12 hours (p = 0.02) between the CD gas and WH gas groups (Fig. 2A and B, Table 6). The odds ratios of a VAS pain score of >30 was 0.16 (95% CI: 0.05, 0.49) at 0 hours and 0.29 (95% CI: 0.10, 0.79) at 12 hours when comparing the WH gas group relative to the CD gas group (Fig. 2A and B, Table 6). No significant difference between the CD gas and WH gas groups were observed at 24 hours (Fig. 2A and B, Table 6).

**Table 6:** Number of patients with a VAS score >30 in the ward.

Hours	IPP				Type of CO_2_ gas			
	12 mmHg		8 mmHg		CD		WH	
	N	N (%)	N	N (%)	N	N (%)	N	N (%)
0	41	31 (76)	41	28 (69)	42	35 (83)	40	24 (60)
4	40	18 (45)	41	14 (34)	42	23 (55)	39	9 (23)
8	41	13 (32)	41	8 (20)	42	13 (31)	40	8 (20)
12	41	11 (27)	41	4 (10)	42	11 (26)	40	4 (10)
16	39	12 (31)	38	4 (10)	40	10 (25)	37	6 (16)
20	34	8 (24)	33	2 (6)	33	4 (12)	34	6 (18)
24	41	10 (24)	41	0 (0)	42	6 (14)	40	4 (10)
48	29	8 (28)	28	1 (4)	31	5 (16)	26	4 (15)

### Intraoperative core temperature

No significant differences in the start, minimum, maximum, mean, or final intraoperative core body temperatures were observed between the CD gas and WH gas groups (Table 2).

### Intraoperative and postoperative complications

One patient in the 8-mmHg with CD gas group had an intraoperative complication (perforation of the stomach by the Veress needle). Postoperatively, the patient had an uneventful recovery and was discharged on postoperative day 3. No patients had postoperative complications.

### Quality of surgical conditions

Scores rated by the surgeon for operative technical difficulty and working space for dissection and suturing were significantly worse in the 8-mmHg group than in the 12-mmHg group (Supplementary Table S1). No significant differences in scores for surgical field visibility were observed between the 8-mmHg group and the 12-mmHg group, or between the CD gas group and the WH gas group Supplementary Table S1). The surgeon experienced low back pain/discomfort during surgery. No significant difference in scores for this pain/physical discomfort rated by the surgeon was observed between the 8-mmHg and 12-mmHg groups (Supplementary Table S1).

## Discussion

Effects of low IPP may be clinically significant for “pain” among the five dimensions of the QoR-40^20^. The present study showed that the odds of a VAS pain score >30 in the ward was over 10 times lower in the 8-mmHg group at 24 hours in agreement with the present analysis of QoR-40 scores. Postoperative pain is correlated with quality of recovery. These findings suggest that low IPP may result in a better quality of postoperative recovery by decreasing postoperative pain. Morphine requirement in the PACU did not differ between the 12-mmHg and 8-mmHg groups. However, the effects did trend in favor of low IPP. Because the power analysis was designed to detect a difference in the gene expression levels, a higher-powered study might have detected a difference in this clinical outcome measure. In the present study, we observed no significant difference in the percentage of patients with VAS pain scores >30 at 24 hours postoperatively between the WH gas group and the CD gas group, which is consistent with the present QoR-40 score analysis. However, we observed that the odds of a VAS pain score >30 was over five times lower in the WH gas group compared to the CD gas group when patients arrived in the ward approximately 2 hours postoperatively. A meta-analysis revealed a statistically significant reduction in VAS pain scores in the WH gas group within 6 hours, but not at 24 hours, postoperatively^21^. Laparoscopic surgery has been considered to cause less postoperative pain than laparotomy. However, one study showed that laparoscopic surgery may be more painful in the immediate postoperative period (0–4 hours) compared to laparotomy^22^. The present findings and a previous meta-analysis^21^ suggest that a WH gas may be effective in reducing pain scores during the immediate postoperative period after laparoscopic surgery. The present molecular analysis indicated that low IPP significantly decreased expression of pro-inflammatory genes (CXCL-2, E-selectin) and significantly increased that of anti-inflammatory genes (IL-10, TSP2) compared to the standard IPP. In addition, low IPP and WH gas significantly increased the HA synthase genes HAS-1 and HAS-2 compared to the standard IPP and CD gas. HA, a major component of the extracellular matrix (ECM), plays a key role in regulating inflammation^23^. HAS-1 and HAS-2 encode high–molecular-weight HA, which suppresses the inflammatory response^23^. These findings suggest that low IPP and WH gas decreased the likelihood of a clinically significant high VAS pain score in the first 24 hours after laparoscopic surgery by reducing inflammation in the surgical laparoscopic peritoneal environment. Further studies to investigate the molecular mechanisms underlying the different effects of low IPP and WH gas on postoperative pain may provide useful information for developing strategies to reduce pain after laparoscopic abdominal surgery. A limitation of the present molecular analysis is that it is unclear whether similar effects of a low IPP and/or WH gas on these 12 genes would be observed, if the surgical procedure was much longer than that in the present study. In addition, it is ethically impossible to perform serial laparoscopies to collect peritoneal biopsy specimens and/or to evaluate the clinical significance of the present molecular findings on clinical outcomes such as postoperative adhesion formation.

A major disadvantage of using a low IPP is that surgical conditions are worse than with the standard IPP. A Cochrane review concluded that the safety of low-pressure pneumoperitoneum has yet to be established^9^. The surgeon’s laparoscopic skills represent a risk factor for perioperative complications of laparoscopic surgery^24^. A major advantage of low IPP is that it requires no additional cost during laparoscopic surgery if the duration of surgery is not affected. The results of the present study and previous studies suggested that experienced surgeons may not need a longer duration of surgery at low IPP even if they realize that surgical conditions may be worse^9^. The present findings may support a clinical benefit of low IPP; however, use of low IPP should be recommended only for skilled, experienced surgeons.

In contrast, use of a WH gas requires no technical effort and could be applied to any abdominal laparoscopic surgery and by any surgeon. In the present study, no significant interactions were observed between IPP and the type of CO_2_ gas used for VAS pain scores. In addition, the present molecular analysis showed that adhesion-formation–related and inflammation-related gene expression levels were less affected when using WH gas at the standard IPP. Thus, when low IPP may not be feasible, use of WH gas at the standard IPP may be a clinically useful alternative for reducing postoperative pain and minimizing adverse effects of a CO_2_ pneumoperitoneum at the standard IPP on the surgical peritoneal environment. However, a major disadvantage of using WH gas is the additional cost. Further studies are required to perform a cost-benefit analysis on the use of WH gas during laparoscopic abdominal surgery.

The present study showed no significant difference in the length of hospital stay between the 12-mmHg IPP and 8-mmHg IPP groups or between the WH gas and CD gas groups. However, length of hospital stay after surgery would be influenced by many different factors such as preoperative counseling regarding expected length of stay, supports in place at home for patient upon discharge and distance from the hospital to home^25, 26^. In the present study, all patients were informed about the expected discharge 3 days after operation.

The external validity of randomized controlled trials (RCTs) is often low^27^. Previous studies have also suggested that a low IPP may reduce postoperative pain compared to the standard IPP^9, 28, 29^. However, almost all of these previous studies were conducted during laparoscopic cholecystectomy;^9, 28, 29^ thus, it is not clear whether the present findings may be generalized to different surgical procedures for other pathologies. In the present study, only patients with a macroscopically normal peritoneum were included. Whether low IPP and/or WH gas would have the same impact on peritoneal gene expression levels and postoperative pain in patients with pathological peritoneum remains to be clarified.

In conclusion, the present randomized 2 × 2 factorial trial showed that low IPP and/or WH gas significantly lowered expression of inflammation-related genes in peritoneal tissues compared to the standard IPP and/or CD gas. Low IPP and WH gas independently decreased the likelihood of a high VAS pain score (>30) after surgery.

## Electronic supplementary material

Supplementary info

## References

[1] Matsuzaki S (2011). Impact of intraperitoneal pressure and duration of surgery on levels of tissue plasminogen activator and plasminogen activator inhibitor-1 mRNA in peritoneal tissues during laparoscopic surgery. Hum. Reprod..

[2] Matsuzaki S (2012). Impact of intraperitoneal pressure of a CO_2_ pneumoperitoneum on the surgical peritoneal environment. Hum. Reprod..

[3] Novitsky YW, Litwin DE, Callery MP (2004). The net immunologic advantage of laparoscopic surgery. Surg. Endosc..

[4] Jayne D (2007). Molecular biology of peritoneal carcinomatosis. Cancer Treat. Res..

[5] Wittich P, Steyerberg EW, Simons SH, Marquet RL, Bonjer HJ (2000). Intraperitoneal tumor growth is influenced by pressure of carbon dioxide pneumoperitoneum. Surg. Endosc..

[6] Bourdel N (2007). Peritoneal tissue-oxygen tension during a carbon dioxide pneumoperitoneum in a mouse laparoscopic model with controlled respiratory support. Hum. Reprod..

[7] Matsuzaki S (2009). Molecular mechanisms underlying postoperative peritoneal tumor dissemination may differ between a laparotomy and carbon dioxide pneumoperitoneum: a syngeneic mouse model with controlled respiratory support. Surg. Endosc..

[8] Matsuzaki S (2010). Carbon dioxide pneumoperitoneum, intraperitoneal pressure, and peritoneal tissue hypoxia: a mouse study with controlled respiratory support. Surg. Endosc..

[9] Gurusamy KS, Vaughan J, Davidson BR (2014). Low pressure versus standard pressure pneumoperitoneum in laparoscopic cholecystectomy. Cochrane Database Syst. Rev..

[10] Volz J (1996). Pathophysiologic features of a pneumoperitoneum at laparoscopy: a swine model. Am. J. Obstet. Gynecol..

[11] Neuhaus SJ, Watson DI (2004). Pneumoperitoneum and peritoneal surface changes: a review. Surg. Endosc..

[12] Erikoglu M, Yol S, Avunduk MC, Erdemli E, Can A (2005). Electron-microscopic alterations of the peritoneum after both cold and heated carbon dioxide pneumoperitoneum. J. Surg. Res..

[13] Rosário MT (2006). Does CO_2_ pneumoperitoneum alter the ultra-structuture of the mesothelium?. J. Surg. Res..

[14] Birch DW (2016). Heated insufflation with or without humidification for laparoscopic abdominal surgery. Cochrane Database Syst. Rev..

[15] Rivoire C (2007). Complete laparoscopic treatment of genital prolapse with meshes including vaginal promontofixation and anterior repair: a series of 138 patients. J. Minim. Invasive Gynecol..

[16] Myles PS, Weitkamp B, Jones K, Melick J, Hensen S (2000). Validity and reliability of a postoperative quality of recovery score: the QoR-40. Br. J. Anaesth..

[17] Herrera FJ, Wong J, Chung F (2007). A systematic review of postoperative recovery outcomes measurements after ambulatory surgery. Anesth. Analg..

[18] Dindo D, Demartines N, Clavien PA (2004). Classification of surgical complications: a new proposal with evaluation in a cohort of 6336 patients and results of a survey. Ann. Surg..

[19] Bodian CA, Freedman G, Hossain S, Eisenkraft JB, Beilin Y (2001). The visual analog scale for pain: clinical significance in postoperative patients. Anesthesiology..

[20] Myles PS (2016). Minimal Clinically Important Difference for Three Quality of Recovery Scales. Anesthesiology..

[21] Sammour, T. *et al*. Systematic review of oxidative stress associated with pneumoperitoneum. *Br*. *J*. *Surg*. **96**, 836–850 (2009). Review. 10.1002/bjs.6651 19591166

[22] Ekstein P (2006). Laparoscopic surgery may be associated with severe pain and high analgesia requirements in the immediate postoperative period. Ann. Surg..

[23] Petrey AC, de la Motte CA (2014). Hyaluronan, a crucial regulator of inflammation. Front. Immunol..

[24] Giger UF (2006). Risk factors for perioperative complications in patients undergoing laparoscopic cholecystectomy: analysis of 22,953 consecutive cases from the Swiss Association of Laparoscopic and Thoracoscopic Surgery database. J. Am. Coll. Surg..

[25] Kelly M, Sharp L, Dwane F, Kelleher T, Comber H (2012). Factors predicting hospital length-of-stay and readmission after colorectal resection: a population-based study of elective and emergency admissions. BMC Health Serv. Res..

[26] Aarts MA (2012). Adoption of enhanced recovery after surgery (ERAS) strategies for colorectal surgery at academic teaching hospitals and impact on total length of hospital stay. Surg. Endosc..

[27] Farrokhyar, F. *et al*. Randomized controlled trials of surgical interventions. *Ann*. *Surg*. **251**, 409–416 (2010). Review. 10.1097/SLA.0b013e3181cf863d 20142732

[28] Hua J, Gong J, Yao L, Zhou B, Song Z (2014). Low-pressure versus standard-pressure pneumoperitoneum for laparoscopic cholecystectomy: a systematic review and meta-analysis. Am. J. Surg..

[29] Özdemir-van Brunschot DM (2016). What is the evidence for the use of low-pressure pneumoperitoneum? A systematic review. Surg. Endosc..

