# Supplementary material for: Effects of low intraperitoneal pressure and a warmed, humidified carbon dioxide gas in laparoscopic surgery: a randomized clinical trial
Source: Sci Rep. 2017 Sep 12;7:11287. doi: 10.1038/s41598-017-10769-1 (PMC5595842; doi:10.1038/s41598-017-10769-1)
Supplement: Supplementary file 1 — Supplementary info [file 41598_2017_10769_MOESM1_ESM.pdf]

**Effects of low intraperitoneal pressure and a warmed, humidified carbon dioxide gas in laparoscopic surgery: a randomized clinical trial**

Sachiko Matsuzaki [1,2,*]

Lise Vernis [3]

Martine Bonnin [3]

Celine Houlle [1]

Aurelie Fournet-Fayard [3]

Giuseppe Rosano [3]

Anne Laure Lafaye [3]

Christian Chartier [3]

Agnes Barriere [3]

Brigitte Storme [3]

Jean-Etienne Bazin [3]

Michel Canis [1, 2]

Revaz Botchorishvili [1, 2]

[1] CHU Clermont-Ferrand, Service de Chirurgie Gynécologique, Clermont-Ferrand, France

[2] Université Clermont Auvergne, Institut Pascal, UMR6602, CNRS/UCA/SIGMA, Clermont-Ferrand, France

[3] CHU Clermont-Ferrand, Service d'Anesthésie Réanimation, Clermont-Ferrand, France

***Corresponding Author:**

Sachiko Matsuzaki

CHU Clermont-Ferrand, CHU Estaing, Chirurgie Gynécologique, 1, Place Lucie et Raymond Aubrac, 63003, Clermont-Ferrand, France (sachikoma@aol.com)

**Supplementary Table S1**: **Quality of surgical conditions rated by the operating surgeon**

| | **IPP** | | | **Type of $CO_2$ gas** | | |
|---|---|---|---|---|---|---|
| **Variable** | **12 mmHg (n=41)** | **8 mmHg (n=41)** | **P-value** | **CD (n=42)** | **WH (n=40)** | **P-value** |
| Operative technical difficulty, mean (SD) | 17 (17) | 27 (15) | **0.007** | | | |
| Working space, mean (SD) | | | | | | |
| For dissection | 83 (11) | 74 (16) | **0.004** | | | |
| For suturing | 85 (9.0) | 73(18) | **<0.001** | | | |
| Surgical field visibility, mean (SD) | 85 (14) | 84 (9) | 0.61 | 85 (9) | 84(14) | 0.51 |
| Pain/physical discomfort, median (IQR) | 10 (11) | 15 (12) | 0.12 | | | |

Operative technical difficulty: 0 = no difficulty, 100 = worst level of difficulty

Working space: 0 = worst level of space, 100 = optimal

visibility: 0 = worst level, 100 = perfect

Pain/physical discomfort: 0 = no pain/physical discomfort, 100 = worst level of pain.

**Study protocol:**

# Impacts of Intraperitoneal Pressure and CO2 Gas on Surgical Peritoneal Environment: a randomized clinical trial

Version: 5, Dated: 21-06-2013

**Sponsor:**

CHU Clermont-Ferrand

**Investigators:**

**Service de Chirurgie Gynécologique, CHU de Clermont-Ferrand**

Revaz Botchorishvili

Celine Houlle

Michel Canis

**Service d'Anesthésie Réanimation, CHU de Clermont-Ferrand**

Lise Vernis

Martine Bonnin

Christian Chartier

Aurelie Fournet-Fayard

Giuseppe Rosano

Anne Laure Lafaye

Agnes Barriere

Brigitte Storme

Jean-Etienne Bazin

**Université d'Auvergne Clermont I**

**Service de Chirurgie Gynécologique, CHU de Clermont-Ferrand**

Sachiko Matsuzaki

**Background**

Laparoscopic surgery technology has evolved dramatically over the past 3 decades, and continues to advance. However, much less attention has been focused on how alteration of the laparoscopic surgical environment might improve clinical outcomes. The laparoscopic surgical environment is closed and thus, factors of the surgical environment such as intraperitoneal pressure (IPP), temperature, and humidity can be modified. Our previous studies suggested that low IPP (8 mmHg) might minimize the adverse impacts of a carbon dioxide (CO2) pneumoperitoneum on the surgical peritoneal environment compared to the standard IPP (12 mmHg) (1, 2). However, our previous studies were not randomized (1, 2). In addition, we did not evaluate whether a low IPP (8 mmHg) could improve clinical outcomes (1, 2). A Cochrane review concluded that no evidence is currently available to support the use of a low-pressure pneumoperitoneum in low–anesthetic-risk patients undergoing elective laparoscopic cholecystectomy (3). Regarding temperature and humidity, previous animal and in vitro experiments demonstrated that a cool and dry CO2 (CD) gas, which we use in a clinical setting, might adversely affect the surgical peritoneal environment (4-7). However, no studies have yet evaluated the impact of the combined use of a warmed, humidified CO2 (WH) gas pneumoperitoneum and low IPP during laparoscopic surgery on the peritoneal environment and postoperative clinical outcomes.

We hypothesize that combined use of low IPP and WH gas may be better for minimizing the adverse impact of a CO2 pneumoperitoneum on the surgical peritoneal environment during laparoscopic abdominal surgery and improving postoperative clinical outcomes, compared to the standard IPP and CD gas.

**Objectives**

The primary objective of this study is to compare the impact of low IPP (8 mmHg) versus standard IPP (12 mmHg), and CD gas versus WH gas, on expression levels of 12 genes (5 adhesion-

formation-related genes, 3 inflammation-related genes, and 4 hyaluronan [HA]-related genes) (1, 2) in peritoneal biopsy specimens. Secondary objectives are to compare the impacts of low IPP versus standard IPP, and CD gas versus WH gas, on the quality of postoperative recovery, postoperative pain, intraoperative core body temperature, and intraoperative and postoperative complications.

**Study Population**

We selected patients who undergo laparoscopic subtotal hysterectomy with promontofixation for uterine prolapse for the following reasons. The duration of this surgery is relatively long among gynecologic endoscopic surgeries, thus enabling us to perform a time-course study of gene expression levels in peritoneal tissues within the same patients. Furthermore, the surgical procedure has been standardized in our department (8); therefore, the results were expected to be minimally influenced by the heterogeneity of surgical interventions and duration of surgery.

Inclusion Criteria:

1. Age 45 to 75 years, menopausal status,
2. American Society of Anesthesiologists (ASA) physical status classification of 1 or 2.

Exclusion Criteria:

1. Refusal to participate
2. Previous history of pelvic surgery for endometriosis and/or infection
3. Pathological peritoneal tissue such as endometriosis, peritoneal adhesions
4. BMI >30
5. Height <150 cm

6. Inability to consent or complete the QoR-40 questionnaire due to cognitive impairment or language barrier.
7. A change in IPP is required during surgery.

**Study design**

Prospective, 2×2 factorial, four–parallel-group, single-center, single-blinded (patients), superiority randomized trial.

**Randomization**

After obtaining informed written consent, participants are allocated in a 1:1:1:1 ratio by a remote 24-hour-a-day computer-generated randomization system hosted at the Institute for Medical Informatics, Statistics and Documentation, Medical University of Graz, in Graz, Austria (https://www.randomizer.at/) using an algorithm with BMI (<25 vs. ≥25), height (<160 vs. ≥160 cm), and age (<65 vs. ≥65 years) as minimization covariates. This randomized trial employed a 2×2 factorial design, with IPP and types of $CO_2$ gas as factors, resulting in four experimental arms: 1) 12-mmHg IPP with CD gas, 2) 12-mmHg IPP with WH gas, 3) 8-mmHg IPP with CD gas, and 4) 8-mmHg IPP with WH gas. After randomization, patients can only be excluded if pathological peritoneal tissues such as adhesions are detected just after insertion of the four trocars, a different surgical technique is needed because the sacral promontory is not clearly identified, IPP is changed during the surgery, conversion to laparotomy, or withdrawal of consent. Enrollment in this study is voluntary and patients are allowed to withdraw at any time.

**Blinding**

Patients are blinded for the allocated treatment arm until the end of the study. The nurse anesthetists in the postanesthesia care unit (PACU) and the ward nurses, who evaluate postoperative pain using a visual analogue scale (VAS), are also blinded. The surgeon can not be blinded, because the surgeon can easily determine whether a low or standard IPP was used. Anesthetists and nurses in the operating room are not blinded for security reasons. All clinical data are collected on anonymous case record forms in a binder by the principal investigators. All peritoneal biopsies samples are identified only by their unique study number. The database is submitted for analysis to independent statisticians who are blinded and are neither involved in the trial management nor employed by the trial sponsor.

**Risks and Benefits**

A number of risks are associated with laparoscopic sub-total hysterectomy with promontofixation for genital prolapse; however, this protocol is expected to add minimal additional risks to the surgical procedure in patients receiving low IPP. Until now, we have observed no complications related to the use of low IPP. This study involved 1 staff surgeon who performe all surgeries with the assistance of a surgical resident. The surgeon has already performed >1,000 cases of the same laparoscopic surgical procedure for genital prolapse and has been applying low IPP (6-8 mmHg) for >15 years without any low-IPP–related intraoperative or postoperative complications. No known benefits for subjects who enroll in the study have been identified. Potential benefits to patients who receive low IPP and/or WH gas exist if we can show that low IPP and/or WH gas can improve postoperative clinical outcomes, compared to a CD gas pneumoperitoneum at the standard IPP.

**Study procedures**

Anesthetic management is performed by 8 staff anesthesiologists and anesthesiology residents or nurse anesthetists under their supervision. In the operating room, standard ASA anesthetic monitors are placed. All patients receive a standardized general anesthetic consisting of premedication with oral administration of 1 mg/kg hydroxyzine hydrochloride suspension 1 hour preoperatively and induction with target-controlled infusion of 0.2 mcg/kg sufentanil and 3 to 5 mg/kg IV propofol. Two mg/kg IV cisatracurium were used to facilitate tracheal intubation. The patient's lungs are ventilated with a 40:60 mixture of oxygen to nitrous oxide. Desflurane and target-controlled infusion of sufentanil are added for maintenance. To assure suitable operating conditions, neuromuscular blockade is maintained using cisatracurium. After induction of anesthesia, all patients receive intraoperative forced-air warming, which is placed on the patient by the anesthesia staff. Intraoperative core temperature is measured at 15-minute intervals using an esophageal probe. For prevention of postoperative nausea and vomiting, 8 mg IV dexamethasone at the beginning of intervention, and 1 mg IV droperidol 1 and 4 mg IV ondansetron at the end of intervention is administrated. 30 minutes before the end of intervention, all patients received 1 g IV paracetamol and 50 mg IV ketoprofene for prevention of postoperative pain.

This study involves 1 staff surgeon who performe all operations with the assistance of a gynecological surgical resident. Insufflation of CO2 gas is performed using a Storz electronic endoflator (Karl Storz Endoscopy & GmbH, Tuttlingen, Germany). CO2 gas is warmed to 37 °C and humidified to 98% RH using the Fisher & Paykel MR860 Laparoscopic Humidification System (HumiGard, Fisher & Paykel Healthcare, Auckland, New Zealand). For the groups receiving WH gas, 180 mL sterile water is added to the chamber and the humidifier was switched on. For the groups receiving standard CD gas, sterile water is not added to the chamber and the humidifier is not switched on, and CO2 gas is delivered at room temperature (21°C) and 0% relative humidity.

When the IPP reached 15 mmHg, four trocars are inserted, immediately after which the IPP is decreased to 12 or 8 mmHg and then maintained at these levels throughout surgery. The duration between

insufflation of $CO_2$ gas and insertion of the four trocars was <5 minutes. For all patients, 5 mL ropivacaine hydrochloride solution (2 mg/mL) is infiltrated around the trocar wounds. All incisions are made after ropivacaine infiltration. In addition, 20 mL ropivacaine solution (2 mg/mL) is infused at the beginning of the operation after pneumoperitoneum creation under the right hemidiaphragm. The local anesthetic infusion is performed using the suction device under visual control. In our clinical setting, we have been systematically performing infusion of ropivacaine solution under the right hemidiaphragm at the beginning of the operation after pneumoperitoneum creation for over 10 years. During this time, we have had no or few complaints about shoulder pain after laparoscopic surgery.

Laparoscopic sub-total hysterectomy with promontofixation used the same surgical technique previously described by our group is performed (8). Macroscopically normal peritoneum is collected from the anterior parietal wall at the beginning of surgery and every 60 minutes thereafter as previously described. The area from which the peritoneal biopsy is acquired is intact and located at a constant distance from the port through which the $CO_2$ gas is insufflated. In view of the fragility of the mesothelial layer, peritoneal biopsies are performed meticulously to minimize the possibility of trauma to the specimens. The full thickness of the peritoneum is excised using only a pair of scissors, and peritoneal tissues are then collected by atraumatic forceps in all cases. Peritoneal samples are immediately collected in RNAlater (Ambion, Cambridgeshire, UK) and stored at -20 °C until further analysis.

On arrival in the PACU, patients are asked to rate their pain at rest using a VAS. After the initial rating, pain ratings are repeated every 20 minutes during the remainder of the PACU stay. When the pain score was >30 of 100, postoperative pain is treated with an IV bolus of 2 to 3 mg morphine, and then 1 to 2 mg IV every 10 minutes to achieve a pain score ≤ 30 of 100. Intravenous patient-controlled analgesia (IV-PCA) is prepared using morphine (1 mg/mL) and droperidol (0.05 mg/mL). PCA devices were programmed with a demand dose of 1 mL (morphine 1 mg) and a lockout period of 10 minutes, with no background infusion.

On arrival in the ward, patients are asked to rate their pain at rest using a VAS. Then, the intensity of postoperative pain at rest and/or on moving is assessed using a VAS every 4 hours until 24 hours postoperatively, then 3 times/day until discharge. All patients receive 1 g paracetamol and 50 mg of ketoprofene IV every 6 hours until 24 hours postoperatively.

Then, pain is managed using oral paracetamol and ketoprofene. If the pain score was ≥3 of 10 at 24 hours postoperatively and if <20 mg IV morphine were administrated within 24 hours, 10 mg oral morphine are administered every 4 hours. The quality of postoperative functional recovery is assessed using the QoR-40 questionnaire (10), which assesses five dimensions of recovery: emotional state (9 items), physical comfort (12 items), physical independence (5 items), psychological support (7 items), and pain (7 items). Each item was rated on a five-point Likert scale: positive items are scored from 1 (worst) to 5 (best); scores were reversed for negative items. The total score on the QoR-40 ranges from 40 (poorest quality of recovery) to 200 (best quality of recovery) (9). The QoR-40 was administered four times, the day before surgery (between 7:00 and 8:00 pm, baseline score), 24 hours and 48 hours postoperatively, and at the first postoperative visit (approximately 30 days after surgery). When patients are discharged from the hospital prior to 48 hours postoperatively, the pain score and QoR-40 are not evaluated at 48 hours. Intraoperative and postoperative complications are recorded and postoperative complications are classified according to the Clavien-Dindo classification (10). Quality of surgical conditions, including the operative technical difficulty, working space, visibility, and pain experienced by the surgeon such as shoulder pain, backache, and hand/finger joint pain during surgery, is rated by the operating surgeon at the end of each procedure using visual analogue scales consisting of 10-cm lines anchored at both ends with 0 and 10 (operative technical difficulty: 0 = no difficulty, 10 = worst level of difficulty; working space: 0 = worst level of space, 10 = optimal; visibility: 0 = worst level, 10 = perfect; and pain/physical discomfort: 0 = no pain/physical discomfort, 10 = worst level of pain).

Total RNA is extracted from peritoneal biopsies using the Qiagen RNeasy Mini Kit according to the manufacturer's instructions (Qiagen, Courtaboef, France) as previously described (1, 2). RNA yield

and integrity are analyzed using the RNA 6000 Pico kit and the Agilent Bioanalyzer 2100 (Agilent Technologies, Santa Clara, CA, USA) as previously described (1, 2). mRNA levels of 12 genes (connective tissue growth factor [CTGF], matrix metalloproteinase-9 [MMP-9], plasminogen activator inhibitor-1 [PAI-1], tissue plasminogen activator [tPA], thrombospondin-2 [TSP-2], chemokine (C-X-C motif) ligand 2 [CXCL-2], E-selectin, interleukin-10 [IL-10], hyaluronic acid synthase-1 [HAS-1], HAS-2, HAS-3, and hyaluronidase-1 [Hyal-1]) were measured by quantitative real-time RT-PCR with a Light Cycler (Roche, Mannheim, Germany) as previously described (1, 2).

**Reporting of Adverse Events or Unanticipated Problems involving Risk to Participants or Others**

The study is designed to be stopped any time if a grade IV or V (using the Clavien-Dindo classification [10]) postoperative complication occurred due to the low IPP. An independent data monitoring committee assesse every 20 recruited patients; if >20 patients can not be recruited within 1 year after the start of the study or if the frequency of severe postoperative complications is >6%, the study is designed to be stopped. If the study is stopped, the study sponsor is required to inform the Consultative Committee for Protection of Persons in Biomedical Research (CPP) of the Auvergne (France) region and the competent French authority (ANSM) within 2 weeks.

**Sample Size**

The power calculation of the present trial is based on our previous studies (1, 2) and our pilot study. The standard deviation is calculated from these gene expression level results for 12 genes and differences that we considered biologically plausible with a significance level of 0.05; 40 patients for each group enable a power of 91-95% for each gene.

**Statistical Methods**

The STATA program version 13.1 (StataCorp, College Station, TX, USA) is used for statistical analysis.

The global QoR-40 scores and the dimensions of the QoR-40 questionnaire are analyzed using the generalized linear mixed model to allow for repeat measurements over time from each patient. The baseline score is considered as a covariate in the analysis, and three main factors are used in the analysis: IPP, type of $CO_2$ gas, and time point.

VAS pain scores were grouped into three categories; ≤ 30, 31–70, and > 70. A study showed that grouping VAS scores into categories (≤ 30, 31–70, and > 70) provides greater clinical relevance for comparisons than using the full spectrum of measured values or changes in value, when pain is an outcome measure in postoperative patients (11). During the PACU stay, the maximum pain score before receiving morphine is assessed at a single time point. Logistic regression is used for the analysis. During the inpatient ward stay, pain scores are measured at multiple time points. To allow for multiple measurements over time, the analysis is performed using mixed logistic regression methods. Two-level models are used, with individual measurements nested within patients. To allow for varying pain scores over time, terms for time are included in the regression model. Linear, squared, and cubic terms for time are all included to allow modelling of a flexible relationship between time and pain score. Interactions between the two treatments (IPP and type of $CO_2$ gas) and time are included to allow the treatment effects to vary over the course of the inpatient stay. The regression models are simplified to omit non-significant interactions from the final model. As with pain score during the PACU stay, scores are analyzed as a categorized binary variable. Mixed logistic regression is used for the analysis.

The gene expression levels in peritoneal biopsy specimens relative to levels of a reference gene (GAPDH) are assessed at 0 hours, and at 1 and 2 hours during a $CO_2$ pneumoperitoneum. This analysis approach considered the 1- and 2-hour values as separate outcomes, which are thus analyzed separately. Linear regression is used for the analysis, with the baseline (0 hours) values include as a covariate.

For the remaining analyses, groups are compared using Fisher's exact test for categorical variables, the Mann-Whitney U test for nonparametric continuous variables, and the t test for parametric continuous variables. Continuous variable parametricity is tested using the Shapiro-Wilk test. Statistical significance is accepted at the 0.05 level. No subgroup analyses are planned.

**References**


1. Matsuzaki S, Botchorishvili R, Jardon K, Maleysson E, Canis M, Mage G. (2011) Impact of intraperitoneal pressure and duration of surgery on levels of tissue plasminogen activator and plasminogen activator inhibitor-1 mRNA in peritoneal tissues during laparoscopic surgery. Hum Reprod, 26:1073-81.
2. Matsuzaki S, Jardon K, Maleysson E, D'Arpiany F, Canis M, Botchorishvili R (2012). Impact of intraperitoneal pressure of a CO2 pneumoperitoneum on the surgical peritoneal environment. Hum Reprod. 27:1613-1623.
3. Gurusamy KS, Vaughan J, Davidson BR (2014). Cochrane Database Syst Rev. CD006930. Low pressure versus standard pressure pneumoperitoneum in laparoscopic cholecystectomy.
4. Volz J, Köster S, Weiss M, Schmidt R, Urbaschek R, Melchert F, Albrecht M. (1996) Pathophysiologic features of a pneumoperitoneum at laparoscopy: a swine model. Am J Obstet Gynecol. 174:132-40.
5. Neuhaus SJ, Watson DI. (2004) Pneumoperitoneum and peritoneal surface changes: a review. Surg Endosc. 18:1316 –22.
6. Erikoglu M, Yol S, Avunduk MC, Erdemli E, Can A. (2005) Electron-microscopic alterations of the peritoneum after both cold and heated carbon dioxide pneumoperitoneum. J Surg Res. 125:73–7.
7. Rosário MT, Ribeiro U Jr, Corbett CE, Ozaki AC, Bresciani CC, Zilberstein B, Gama-Rodrigues JJ. (2006) Does CO2 pneumoperitoneum alter the ultra-structuture of the mesothelium? J Surg Res. 133:84–8.
8. Rivoire C, Botchorishvili R, Canis M, Jardon K, Rabischong B, Wattiez A, Mage G. (2007) Complete laparoscopic treatment of genital prolapse with meshes including vaginal promontofixation and anterior repair: a series of 138 patients. J Minim Invasive Gynecol. 14:712-8.
9. Myles PS, Weitkamp B, Jones K, Melick J, Hensen S. (2000) Validity and reliability of a postoperative quality of recovery score: the QoR-40. Br J Anaesth. 84:11-5.
10. Dindo D, Demartines N, Clavien PA. (2004) Classification of surgical complications: a new proposal with evaluation in a cohort of 6336 patients and results of a survey. Ann Surg. 240:205-13.
11. Bodian, C.A., Freedman,G., Hossain, S., Eisenkraft, J.B., & Beilin, Y. The visual analog scale for pain: clinical significance in postoperative patients. Anesthesiology. **95**:1356-1361(2001).
